# Effectiveness of COVID-19 Vaccination in Preventing All-Cause Mortality among Adults during the Third Wave of the Epidemic in Hungary: Nationwide Retrospective Cohort Study

**DOI:** 10.3390/vaccines10071009

**Published:** 2022-06-24

**Authors:** Anita Pálinkás, János Sándor

**Affiliations:** Department of Public Health and Epidemiology, Medical Faculty, University of Debrecen, H-4028 Debrecen, Hungary; janos.sandor@med.unideb.hu

**Keywords:** COVID-19 vaccination, general mortality, vaccine cohorts, healthy vaccinee effect, real-life vaccine effectiveness

## Abstract

Our investigation aimed to describe the all-cause mortality rates by COVID-19 vaccination groups in Hungary for an epidemic period (1 April 2021–20 June 2021) and a nonepidemic period (21 June 2021–15 August 2021), and to determine the vaccines’ effectiveness in preventing all-cause mortality utilizing nonepidemic effectiveness measures to adjust for the healthy vaccinee effect (HVE). Sociodemographic status, comorbidity, primary care structural characteristics, and HVE-adjusted survival difference between fully vaccinated and unvaccinated cohorts in the epidemic period had been computed by Cox regression models, separately for each vaccine (six vaccines were available in Hungary). Hazard ratio (HR) reduction in epidemic period corrected with nonepidemic period’s HR with 95% confidence interval for each vaccine was used to describe the vaccine effectiveness (VE). The whole adult population (N = 6,404,702) of the country was followed in this study (4,026,849 fully vaccinated). Each vaccine could reduce the HVE-corrected all-cause mortality in the epidemic period (VE_Oxford/AstraZeneca_ = 0.592 [0.518–0.655], VE_Janssen_ = 0.754 [0.628–0.838], VE_Moderna_ = 0.573 [0.526–0.615], VE_Pfizer-BioNTech_ = 0.487 [0.461–0.513], VE_Sinopharm_ = 0.530 [0.496–0.561], and VE_Sputnik_ _V_ = 0.557 [0.493–0.614]). The HVE-corrected general mortality for COVID-19 vaccine cohorts demonstrated the real-life effectiveness of vaccines applied in Hungary, and the usefulness of this indicator to convince vaccine hesitants.

## 1. Introduction

By 20 December 2021, there were 5,354,794 confirmed cases of COVID-19 death registered worldwide [1]. Because of the limited effectiveness and/or unbearable socioeconomic cost of precautionary actions, intensive testing for isolating those infected, and lockdown measures, mass vaccination seems to be the most important element for stopping the COVID-19 pandemic [2]. However, vaccines with proven efficacy became available at the beginning of 2021 [3] and have yet to stop the pandemic. This is mainly because of the limited coverage of vaccination. The availability of vaccines against COVID-19 are limited only in low-income countries nowadays (lower than 20% of people have received at least one dose), while the unfavorable coverage of the developed countries can be explained mainly by vaccine hesitancy [4,5]. The acceptance of vaccination is primarily explained by the lack of and/or incorrect knowledge about the COVID-19 pandemic and the effectiveness of the vaccine. There are remarkable differences in the level of knowledge of the population depending on environmental (including policy and media as well) and individual characteristics (e.g., gender, age, ethnicity, income, lifestyle, etc.). The uncertainty of the vaccines’ safety, the potential side-effects (mainly due to the rapid development of vaccines), and the misinformation and conspiratorial beliefs about the severity of COVID-19 are the most listed reasons for vaccine hesitancy [6,7,8,9,10,11,12]. Therefore, it is essential to have valid, simple, and comprehensive messages on the effectiveness of preventative measures. Both the formulation of the pandemic control policy and the organization of mass vaccination require monitoring and demonstration of the vaccination programs’ effectiveness.

COVID-19-caused mortality is the most important effectiveness indicator in addition to the prevalence of infection and vaccination coverage. The real-world effectiveness of COVID-19 vaccines has been demonstrated in terms of COVID-19 mortality reduction as well [13,14]. Mortality statistics are widely understandable; therefore, they can be used in pandemic-related communication to establish the collaboration of stakeholders necessary to prevent population-level health losses. Unfortunately, the cause-of-death diagnosis is subject to some uncertainties, and the quality of COVID-19 death indicators varies across countries. Because all-cause mortality by vaccination status [15,16,17] is not affected by the cause-of-death diagnostic process, it can be a useful, less biased, but less specific indicator for routine population-level monitoring. The obvious problem is that a comparison between vaccinated and unvaccinated groups presents together the impact of the vaccine and the consequence of the difference in vaccine-independent risk factor patterns. The experience is that health consciousness, socioeconomic status, and health care use vary profoundly by vaccination status in the case of voluntary vaccinations [18]. Due to the healthy vaccine effects [18,19,20], the all-cause mortality difference can overestimate the health gain achieved by vaccines. Moreover, zero health gain by vaccination cannot be excluded when interpreting this difference. In fact, there are 15 countries using routine all-cause mortality by vaccination monitoring [21].

Vaccine availability and the publication of safety and efficacy reports determined the timing of vaccine introduction into the Hungarian vaccination program (26/12/2020 Pfizer-BioNTech COVID-19 mRNA vaccine [22]; 16 January 2021 Moderna COVID-19 mRNA-1273 vaccine [23]; 9 February 2021 Oxford/AstraZeneca COVID-19 vaccine [24]; 11 February 2021 Sputnik V vaccine [25]; 23 February 2021 Sinopharm COVID-19 vaccine [26]; 5 May 2021 Janssen Ad26.COV2) [15]. Target vaccination groups defined by age, chronic disease status, occupation, pregnancy, and nursing home residence varied in timing because they were tailored to the actual pandemic situation. Due to these, the vaccination rate in Hungary is similar to the European Union average [4].

Mass vaccination was implemented by COVID-19 vaccination centers established in secondary institutions and by general practitioners (GPs). GPs had to organize vaccination-related tasks in parallel with the maintenance of primary care adapted to pandemic circumstances. Because the quality of primary care varied by the structural characteristics of the general medical practices (GMPs) in the prepandemic period [27,28,29], the quality of this adapted care probably operated with variability in the pandemic period as well.

Despite the adequate availability of vaccines and the average vaccination coverage, according to the European Centre for Disease Prevention and Control (ECDC), the cumulative COVID-19 mortality rate in Hungary (377/100,000) is the fifth highest in the world [30], suggesting that Hungarian pandemic management is critical. However, the excess mortality in Hungary does not deviate from the European average [31], demonstrating that the Hungarian cause of death ascertainment practice (all cases of death with a recent positive COVID-19 test result are considered COVID-19-caused death ir-respective of the comorbidities and the real process that led to death) omits the pathological evaluation of the causal role of SARS-CoV-2 infection [32]. This suggests the opposite evaluation of crisis management. Vaccination-specific all-cause mortality-based indicators have not yet been used in Hungary, but mortality monitoring became feasible from 1 April 2021, when the third wave reached its peak.

Our investigation aimed to describe the all-cause mortality rates adjusted for sociodemographic status and comorbidities of patients and structural characteristics of GMPs among fully vaccinated and unvaccinated adults in Hungary during an epidemic (1 April 2021–20 June 2021) and a nonepidemic period (21 June 2021–15 August 2021). Our further goal was to determine the effectiveness of each applied vaccine in preventing mortality, correcting the epidemic period’s mortality rates with the healthy vaccinee effect quantified by the mortality rates observed in the nonepidemic period.

## 2. Materials and Methods

### 2.1. Setting

The third wave of COVID-19, dominated by the Delta variant of SARS-CoV-2, launched in January 2021 and terminated in August 2021 in Hungary. Data were available for the mortality monitoring project from 1 April 2021, when the expected protective effect of administered vaccines was expected to become detectable.

The State Secretary of Health ensured the availability of the database with unidentified, person-level data for our retrospective cohort investigation. There was no primary data collection in this study; we used routinely collected and continuously available health care-related data. There is only one health insurance company in Hungary which registers the processes of health care interventions. Therefore, the data collection is based on standardized tools and methods, and the database covered the whole population of the country. (Voluntary health insurance represents a negligible proportion of Hungarian health care financing [33].) The database is constructed with the agreement of Medical Chief Officer (19448-2/201/NSEF) in order to evaluate survival by patient subgroups while controlling for comorbidities, demographic status, and structural characteristics of GMPs. Both COVID-19 vaccination and death were included in this dataset as well.

A cohort of 7,495,914 people was established, consisting only of adults at least 18 years of age on 1 April 2021 in Hungary. Excluding records with internal inconsistency (N = 831) and young adults who were seen at a pediatric general medical practice (57,117), the resulting database consisted of 7,437,966 records. In this cohort, we evaluated the association between vaccination status and all-cause mortality from 1 April 2021 to 15 August 2021. The investigated period was divided into 2 parts based on the number of verified infections and the daily number of deaths. The epidemic period was defined between 1 April 2021 and 20 June 2021, while the second part of the follow-up time (from 21 June 2021 to 15 August 2021) was considered as a nonepidemic period. During these periods, the number of verified infections per day was 1984 and 51, respectively, while the daily number of deaths was 91 and 1, respectively (epidemic curve in Supplementary Materials Figure S1) [30].

### 2.2. Vaccination Status

Six different vaccines were applied in Hungary during the studied period (Janssen (Johnson and Johnson, New Brunswick, NJ, USA), Moderna (National Institute of Allergy and Infectious Diseases and Biomedical Advanced Research and Development Authority, Cambridge, MA, USA), AstraZeneca (Oxford University, Oxford, UK), Pfizer (BioNTech, Mainz, Germany), Sinopharm (Wuhan Institute of Biological Products, Wuhan, China), and Sputnik (Gamaleya National Research Centre of Epidemiology and Microbiology, Moscow, Russia)). Adults were considered fully vaccinated 7 days after the completion of vaccination (administration of the first dose of the Janssen vaccine or the second dose of other vaccines), and these individuals were further grouped into 6 different cohorts according to the type of vaccine. Individuals were classified as unvaccinated until the first dose of the COVID-19 vaccine. Partially vaccinated people who received the vaccine during the follow-up period but did not become protected during the study period (did not receive the booster or died before the 7th day after complete vaccination) were excluded from the investigation.

### 2.3. Studied Variables

The date of death was registered as inactivation of health insurance identifier by death of patient. The sociodemographic confounding factors of the study model were defined as follows: age on 1 April 2021 by year, sex, and exemption certificate eligibility (the local municipality can issue an exemption certificate as a social benefit for deprived patients with chronic disease if it is recommended by the patient’s GP). GMPs seeing adults were classified by list size (≤800 patients, 801–1200 patients, 1201–1600 patients, 1601–2000 patients, and >2000 patient categories), settlement type (distinguishing urban and rural GMPs), and geographical location by county. The GP vacancy and the age and sex of the GP could also be inserted into the statistical models (making the distinction between GMPs with GPs <65 years old male, <65 years old female, ≥65 years old male, ≥65 years old female, and GP vacancy with GP replacement by temporary contract), because in Hungary, the overwhelming majority of GMPs belong to one GP; solo practices are typical. The relative education (the number of years of school attendance) and the relative employment for GMP were determined on the basis of 2011 Hungarian Census data. The settlement-specific data were standardized by age and sex. Using the distribution of patients’ places of living, the standardized relative education and standardized relative employment were calculated for each GMP.

The comorbidities were ascertained by outpatient and hospital discharge records, reports from GMPs, and drug consumption records. Diabetes mellitus was indicated by at least 4 redemptions of drugs with an A10 ATC code in the previous 12 months. Ischemic heart disease was specified by at least 4 redemptions of drugs with a C07 ATC code or by treatment for acute myocardial infarction or implementation of coronary artery bypass graft surgery or percutaneous transluminal coronary angioplasty in the previous 12 months. Chronic obstructive pulmonary disease (COPD) was defined as at least 3 redemptions of drugs with an R03 ATC code in the previous 12 months or by a J44 ICD10 code in any outpatient discharge record. Hypertension was specified by at least 4 redemptions of drugs with C02, C03, C04, C05, C07, C08, or C09 ATC codes in the previous 12 months. Adults with at least one admission to the hospital with a J45 ICD10 code in the previous 12 months were considered asthmatic. Adults with at least 2 outpatient or inpatient discharge records with C-group ICD10 codes in the previous 12 months were classified as oncologic patients. End-stage renal disease was indicated by at least 12 recorded treatments (acute hemodialysis, chronic hemodialysis, hemofiltration, high flux dialysis, mobile dialysis, peritoneal dialysis, hemodiafiltration, or hemoperfusion) or by at least 1 outpatient or inpatient discharge record with a renal failure ICD10 code (N1800, N1880, N1890) in the previous 12 months. Chronic liver disease was defined as at least 1 outpatient or inpatient discharge record with alcoholic fatty liver (K70 ICD10 code), hepatic failure (K72 ICD10 code), chronic hepatitis (K73 ICD10 code), or fibrosis and cirrhosis of the liver (K74 ICD10 code) in the previous 12 months.

### 2.4. Statistical Analysis

The data of the epidemic and nonepidemic period were analyzed separately. The cohort was divided into unvaccinated and fully vaccinated parts. Adults who became fully vaccinated in the study period were considered unvaccinated until the first dose of the COVID-19 vaccine and fully vaccinated from the 7th day after the completion of vaccination. The fully vaccinated group was further classified by the type of vaccine. The observed time with partial vaccination was not investigated by the statistical models.

Cox regression with time-dependent covariant models was used to quantify the difference between the survival of unvaccinated and fully vaccinated adults. Distinct models were applied for each vaccine using the same unvaccinated cohort as the control. The same set of confounding factors was controlled for each model. Adjusted hazard ratios (HRs) with 95% confidence intervals (CI) were used to describe the association between survival and explanatory variables.

HRs were computed separately for the epidemic (HR_E_) and nonepidemic periods (HR_NE_). The SARS-CoV-2 virus circulation was negligible between 21 June 2021 and 15 August 2021 (as indicated by number of infections), and the observed mortality rates during this period were independent of the protective effect of the vaccination. HR_NE_ represented the survival difference between people who took and did not take the vaccine, independent of the COVID-19-caused risk. The difference between epidemic and nonepidemic HRs (ΔHRs) as a proxy measure of effect size was calculated to estimate the vaccine-attributable difference between the survival of vaccinated and unvaccinated subjects. The vaccine effectiveness (VE) in preventing all-cause mortality was describe with 1 − ΔHRs separately for each vaccine type.

SPSS version 28 (IBM Corporation, New York, NY, USA) was used for the data analysis.

### 2.5. Ethical Approval

Because all data in our secondary analyses were unidentified and information potentially connectable to individuals was not utilized, informed consent was not required according to Hungarian legislation, as it was acknowledged by the Ethics Committee of the Hungarian National Scientific Council (BMEÜ/279/2022/EKU).

## 3. Results

Because the partially vaccinated adults (epidemic period: N = 1,033,264; nonepidemic period: N = 189,568) were excluded from the analyses, the analyzed cohort size was 6,404,702 in the epidemic period and 7,215,790 in the nonepidemic period.

Vaccination coverage was 9.02% (577,927/6,404,702) at the beginning, 56.09% (4,047,638/7,215,790) on 21 June 2021, and 69.17% (4,979,306/7,198,937) at the end of the observation period. Sociodemographic composition, comorbidity pattern, and GMP structural indicators showed great variability across vaccination groups, reflecting the differences in the recommended target population for investigated vaccines (The detailed data on the composition of the sample are summarized in the Supplementary Materials Tables S1–S5).

### 3.1. Epidemic Period

The population was composed of 4,026,849 fully vaccinated and 2,377,853 unvaccinated adults in the epidemic period. The Pfizer vaccine (N = 1,604,250) was used most frequently, followed by Sinopharm (N = 919,610), Sputnik (N = 847,698), AstraZeneca (N = 333,480), Moderna (N = 243,416), and Janssen (N = 78,395) (Tables S1 and S2).

**Figure 1:**
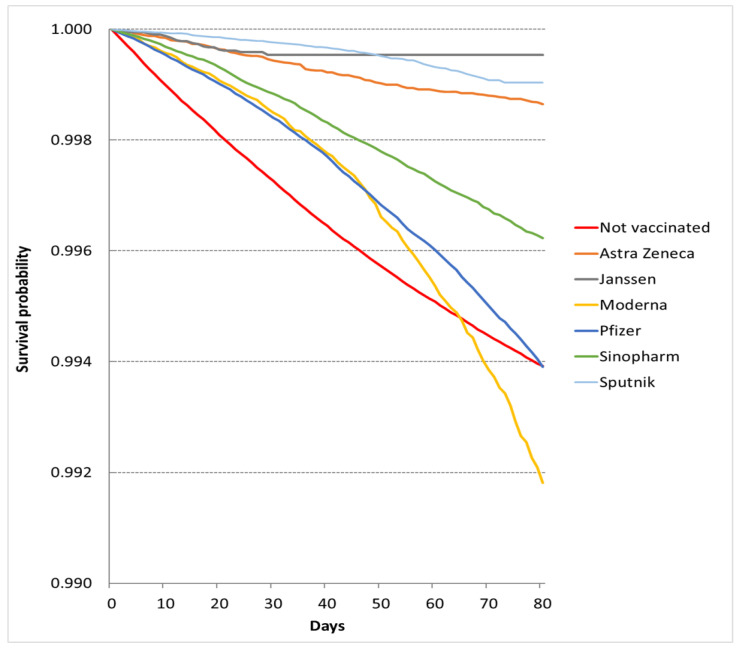
Survival probabilities in vaccination cohorts in the epidemic period.

Altogether, 26,430 deaths were registered from 1 April 2021 to 20 June 2021. The number of deaths and the observed time were 8043 and 173,168,589 person-days among fully vaccinated individuals and 18,387 and 229,492,866 person-days among unvaccinated individuals. The crude mortality rate was 4.64/100,000 person-days (95% CI 4.55–4.74/100,000 person-days) among fully vaccinated patients and 8.01/100,000 person-days (95% CI 7.90–8.12/100,000 person-days) among unvaccinated patients. Remarkable variation was observed in vaccine-specific crude mortality rates (Table 1). The crude survival probability was better in each vaccine group than in the unvaccinated cohort apart from the Moderna cohort (Figure 1). The better survival in each vaccine cohort was demonstrated by the adjusted HRs from multivariable Cox models: AstraZeneca 0.129 (95% CI 0.111–0.150), Janssen 0.174 (95% CI 0.118–0.255), Moderna 0.187 (95% CI 0.173–0.202), Pfizer 0.197 (95% CI 0.190–0.203), Sinopharm 0.147 (95% CI 0.140–0.154), and Sputnik 0.098 (95% CI 0.088–0.108) (Table 2).

Higher age, male sex, eligibility for exemption certificate, and each of the investigated chronic diseases proved to be significant person-level risk factors. The structural indicators of GMPs showed no consequent association with survival, but significant geographical variability could be observed for each vaccine group, and the relative education of people belonging to a GMP proved to be a significant protective factor for the Moderna, Pfizer, and Sinopharm cohorts (Table S5).

### 3.2. Nonepidemic Period

After excluding the partially vaccinated people, 4,989,611 adults were fully vaccinated and 2,226,179 people were not vaccinated in the investigated cohort between 21 June 2021 and 15 August 2021. The order of the received vaccines was the same as during the epidemic period: the Pfizer vaccine was used the most frequently and the Janssen was the least common vaccine, with usage by 29.5% and 1.4% of the adults, respectively (Tables S3 and S4).

**Figure 2:**
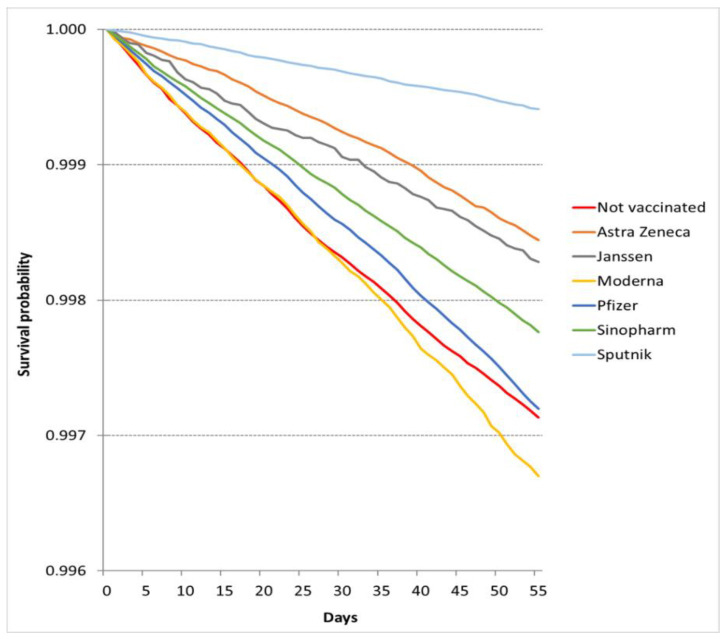
Survival probabilities in vaccination cohorts in the nonepidemic period.

In the nonepidemic period, the number of deaths was 16,853. The crude mortality rate was 3.98/100,000 person-days (95% CI 3.91–4.06/100,000 person-days) among fully vaccinated patients and 5.32/100,000 person-days (95% CI 5.20–5.45/100,000 person-days) among unvaccinated patients (Table 3). The observed survival probabilities were better in the unvaccinated group than in the Moderna-vaccinated groups (Figure 2), but the multivariate models showed better survival for each vaccination group (Table 2). The COVID-19 independent risk of death was smallest in the Sputnik cohort (HR_NE_: 0.221, 95% CI 0.202–0.242), followed by the Sinopharm (HR_NE_: 0.312, 95% CI 0.297–0.328), AstraZeneca (HR_NE_: 0.317, 95% CI 0.294–0.341), Pfizer (HR_NE_: 0.384, 95% CI 0.370–0.399), Moderna (HR_NE_: 0.438, 95% CI 0.409–0.469), and Janssen (HR_NE_: 0.707, 95% CI 0.604–0.828) cohorts in the nonepidemic period. (The descriptive statistics and Cox models are summarized in Supplementary Materials Tables S3, S4 and S6 ).

### 3.3. Vaccine Effectiveness (VE) in Preventing All-Cause Mortality

Because the HRs for the epidemic-free period represented the difference in survival due to healthy vaccinee effect, the epidemic HRs corrected with nonepidemic HRs were used to describe the effectiveness of vaccines in preventing all-cause mortality. Our results demonstrated that each vaccine improved the survival probabilities. In this respect, the Pfizer vaccine (VE: 0.513, 95% CI 0.487–0.539) showed a weaker protective effect than the other vaccines (Janssen VE: 0.246, 95% CI 0.162–0.372; AstraZeneca VE: 0.408, 95% CI 0.345–0.482; Moderna VE: 0.427, 95% CI 0.385–0.474; Sputnik VE: 0.443, 95% CI 0.386–0.507; Sinopharm VE: 0.470, 95% CI 0.439–0.504) (Table 2).

## 4. Discussion

### 4.1. Main Findings

Although several studies confirm the real-world effectiveness of the six different COVID-19 vaccines available in Hungary [13,34], according to our best knowledge, neither vaccine had been evaluated in the respect of the real-effectiveness on all-cause mortality controlling for the healthy vaccinee effect. This nationwide retrospective cohort study investigated all-cause mortality in the general adult population among unvaccinated individuals and those vaccinated with one of six different COVID-19 vaccines in Hungary in the epidemic period of 1 April 2021–20 June 2021 and the nonepidemic period of 21 June 2021–15 August 2021. Because the survival adjusted for the presence of chronic diseases, sociodemographic indicators, and characteristics of primary care services differed significantly between the vaccine cohorts in the nonepidemic period, this analysis could demonstrate that the pattern of uncontrolled confounding factors (e.g., smoking, obesity, seriousness of the included chronic diseases, chronic diseases not investigated, patients’ attitudes toward collaborating with health care services) varied across the vaccinated and unvaccinated cohorts. In the case of voluntary vaccinations, such as the COVID-19 vaccine, the correction of these factors is essential to evaluate the effectiveness of the vaccine, otherwise the real protective effect will be overestimated.

The observed results adjusted for chronic diseases, sociodemographic indicators, characteristics of primary care services, and healthy vaccinee effects showed that survival improved in each vaccinated cohort among adults in the epidemic period. In fact, the AstraZeneca, Janssen, Moderna, Pfizer, Sinopharm, and Sputnik vaccinations were associated with 59.2%, 75.4%, 57.3%, 48.7%, 53.0%, and 55.7% effectiveness against all-cause death in the studied epidemic period.

It cannot be excluded that the observed variability in mortality reduction is attributable to the variability of uncontrolled confounding factors in the vaccine cohorts. For example, it was shown by our investigation that the Sputnik and Janssen vaccines were preferred among younger adults with advantageous social status, while Pfizer was preferred among older adults with chronic diseases. Therefore, it is also highly probable that Pfizer was preferred among patients with diseases not controlled for in our investigation, and adults with better social status were less exposed to chronic diseases not controlled for in our investigation.

Underlying diseases and sociodemographic factors had roles as expected. To emphasize an obvious fact, without adjusting for these factors, the all-cause mortality analysis is meaningless.

The results of previous domestic research have shown that the quality of primary care varied by the structural characteristics of GMPs in the prepandemic period [27,28,29]; therefore, we hypothesized the same during the pandemic period. However, GMP attributes had no remarkable role, suggesting that adjustment for these factors is not important in general mortality monitoring.

### 4.2. Strengths and Limitations

The strengths of the applied design were based on a long observation time for each cohort, which ensured high statistical power. Furthermore, the lack of classification bias for outcome ascertainment, controlling for important risk factors for the studied outcome, adjustment for the healthy vaccinee effect, and the standardized countrywide data collection system for confounding factor assessment also contributed to the validity of our investigation.

Since the composition of the cohorts hardly changed in the nonepidemic period, the confounding pattern in the cohorts was quite stable. The adjustment for the healthy vaccinee effect to control for noninvestigated confounding factors was based on cohorts stable in the respect of the presence of each confounding factor.

The main limitation of our approach was that many confounding factors were not controlled for, which prevented the comparability of the vaccines with each other. Some known prognostic factors (mentioned above) were not available in the analyzed database (these kind of data are not covered by any routine data collection in Hungary), and there are probably unknown prognostic factors [35] as well. Another problem is that as a result of epidemic measures, the pattern of several risk factors (e.g., economic status, smoking, physical activity, diet habits, mental health, access to health care) has changed unfavorably [6,36,37,38,39]. Taking the data on these factors and their change into account would certainly improve the usefulness of the all-cause mortality-based indicators.

Further limitations were that the nonepidemic period was not fully free of virus circulation, which caused a downward bias of the estimations for vaccine effectiveness, and the lack of control for the harvesting effect (mortality replacement) resulted in an overestimation of vaccine effectiveness [40].

The cause of death was not restricted to COVID-19 death in this investigation. On the one hand, the serious discrepancy between Hungarian COVID-19 mortality and excess mortality for the pandemic in Hungary demonstrates that the Hungarian cause-of-death diagnosis for COVID-19 deviates remarkably from the practice of other countries. On the other hand, although it was compulsory to test patients for SARS-CoV-2 infection upon hospital admission, the testing frequency among nonhospitalized adults was exceptionally low (15.4% and 53.1% during the epidemic period and 4.0% and 29.7% during the nonepidemic period in Hungary and the European Union, respectively) [30]. Therefore, the real number of infections and the role of SARS-CoV-2 in nonhospital deaths were not well-known in the studied cohorts.

### 4.3. Implications

Knowing the obvious importance of vaccination in stopping the COVID-19 pandemic [41] and facing the variable but limited coverage of vaccination programs even in developed countries, indicators that could demonstrate the gains achieved by vaccines have a pivotal role. Although vaccine cohort mortality rates are regularly reported in 15 countries, these are not among the vaccine gain indicators of the ECDC, WHO, and CDC [42,43,44].

Our findings demonstrate that the careful approach of high-level institutions is justified since the nonadjusted mortality rates can be misleading. In fact, the crude mortality rates can be higher among vaccinated individuals in epidemic periods than in nonepidemic periods. (Excess mortality in the pandemic period was −1.06, −1.58, 1.25, 1.59, 0.37, −0.09, and 2.69 per 100,000 person-days among the AstraZeneca, Janssen, Moderna, Pfizer, Sinopharm, Sputnik, and unvaccinated cohorts, respectively.)

However, our observations could also demonstrate that confounding factor-adjusted and healthy vaccinee effect-corrected mortality measures can be used to estimate the gain achieved by vaccines with proven efficacy. Taking into account the simplicity of the data collection and the easy-to-understand nature of these indicators, the age-, sex-, eligibility exemption certificate-, education-, comorbidities-, and geographical location-adjusted versions of vaccine effectiveness proposed by our investigation could be used in vaccination program monitoring and in organizing the necessary communication support, even in settings where the COVID-19-cause-of-death diagnosis is uncertain. With explicit admission, these indicators cannot provide evidence for vaccine efficacy [45].

## 5. Conclusions

Our retrospective cohort investigation demonstrated the huge variability of survival across cohorts by COVID-19 vaccination status during a period when the COVID-19 epidemic was not active, meaning the vaccine could not exert a protective effect. The observed differences were not attributable to the sociodemographic characteristics of patients, the presence of the most prevalent underlying diseases, or the structural characteristics of GMPs providing care for patients.

The vaccine effectiveness measures for the epidemic period adjusted by the nonepidemic period measures could demonstrate better survival in each vaccinated cohort than in the unvaccinated cohort.

This study suggests that the all-cause mortality rate by vaccination status is a useful indicator that can support the organization of epidemic control, but by applying a more complete predictor set of lethal COVID-19 outcomes, the usefulness of this indicator could be improved.

## Figures and Tables

**Table 1:** Mortality rate among adults in Hungary by vaccination status between 1 April 2021 and 20 June 2021.

	Deaths, N (%)	Censored, N (%)	Total, N (Vaccine Share %)	Observed Person-Days	Mortality Rate Per 100,000 Person-Days
AstraZeneca	176 (0.05%)	333,304 (99.95%)	333,480 (5.21%)	10,094,843	1.74% [1.49–2.00%]
Janssen	26 (0.03%)	78,369 (99.97%)	78,395 (1.22%)	1,638,877	1.59% [0.98–2.19%]
Moderna	684 (0.28%)	242,732 (99.72%)	243,416 (3.80%)	9,304,244	7.35% [6.82–7.88%]
Pfizer	4804 (0.30%)	1,599,446 (99.70%)	1,604,250 (25.05%)	70,949,254	6.77% [6.59–6.96%]
Sinopharm	1973 (0.21%)	917,637 (99.79%)	919,610 (14.36%)	43,526,230	4.53% [4.34–4.73%]
Sputnik	380 (0.04%)	847,318 (99.96%)	847,698 (13.24%)	37,655,141	1.01% [0.91–1.11%]
*p*-value *	<0.001		-	-	-
Fully vaccinated	8043 (0.20%)	4,018,806 (99.80%)	4,026,849 (62.87%)	173,168,589	4.64% [4.55–4.74%]
Not vaccinated	18,387 (0.77%)	2,359,466 (99.23%)	2,377,853 (37.13%)	229,492,866	8.01% [7.90–8.12%]
*p*-value **	<0.001		-	-	-
Total	26,430 (0.41%)	6,378,272 (99.59%)	6,404,702 (100%)	402,661,455	6.56% [6.49–6.64%]

* χ^2^-test by type of vaccine; ** χ^2^-test comparing vaccinated and not vaccinated subjects.

**Table 2:** Effectiveness of vaccines in preventing all-cause mortality in the period of 1 April 2021 to 15 August 2021 by multivariable Cox regression models.

	HR * for 1 April 2021 to 20 June 2021 Period	HR * for 21 June 2021 to 15 August 2021 Period	Difference of Hazard Ratios	Effectiveness in Preventing All-Cause-Mortality
AstraZeneca	0.129 [0.111–0.150]	0.317 [0.294–0.341]	0.408 [0.345–0.482]	0.592 [0.518–0.655]
Janssen	0.174 [0.118–0.255]	0.707 [0.604–0.828]	0.246 [0.162–0.372]	0.754 [0.628–0.838]
Moderna	0.187 [0.173–0.202]	0.438 [0.409–0.469]	0.427 [0.385–0.474]	0.573 [0.526–0.615]
Pfizer	0.197 [0.190–0.203]	0.384 [0.370–0.399]	0.513 [0.487–0.539]	0.487 [0.461–0.513]
Sinopharm	0.147 [0.140–0.154]	0.312 [0.297–0.328]	0.470 [0.439–0.504]	0.530 [0.496–0.561]
Sputnik	0.098 [0.088–0.108]	0.221 [0.202–0.242]	0.443 [0.386–0.507]	0.557 [0.493–0.614]

* hazard ratio [95% confidence interval] adjusted for age, sex, eligibility for exemption certificate, presence of chronic diseases (diabetes mellitus, ischemic heart disease, COPD, asthma, hypertension, cancer, chronic liver disease, end-stage renal disease), and structural characteristics of the GMP providing the investigated adults (settlement type, location by county, list size, and vacancy of GMP; relative education and employment of the population belong to the GMP, and the age and sex of GP).

**Table 3:** Mortality rate among adults in Hungary by vaccination status between 21 June 2021 and 15 August 2021.

	Deaths, N (%)	Censored, N (%)	Total, N (Vaccine Share %)	Observed Person-Day	Mortality Rate per 100,000 Person-Days
AstraZeneca	792 (0.13%)	592,609 (99.87%)	593,401 (8.22%)	28,240,807	2.80% [2.61–3.00%]
Janssen	160 (0.16%)	98,394 (99.84%)	98,554 (1.37%)	5,044,716	3.17% [2.69–3.66%]
Moderna	1027 (0.32%)	320,948 (99.68%)	321,975 (4.46%)	16,822,495	6.10% [5.74–6.47%]
Pfizer	5603 (0.26%)	2,123,839 (99.74%)	2,129,442 (29.51%)	108,239,884	5.18% [5.04–5.31%]
Sinopharm	2203 (0.22%)	977,239 (99.78%)	979,442 (13.57%)	52,933,464	4.16% [3.99–4.33%]
Sputnik	520 (0.06%)	866,277 (99.94%)	866,797 (12.01%)	47,374,813	1.10% [1.00–1.19%]
*p*-value *	<0.001		-	-	-
Fully vaccinated	10,305 (0.21%)	4,979,306 (99.79%)	4,989,611 (69.15%)	258,656,179	3.98% [3.91–4.06%]
Not vaccinated	6548 (0.29%)	2,219,631 (99.71%)	2,226,179 (30.85%)	123,049,996	5.32% [5.20–5.45%]
*p*-value **	<0.001		-	-	-
Total	16,853 (0.23%)	7,198,937 (99.77%)	7,215,790 (100%)	381,706,175	4.42% [4.35–4.48%]

* χ^2^-test by type of vaccine; ** χ^2^-test comparing vaccinated and not vaccinated subjects.

## Data Availability

Data that support the findings of this study are available from the corresponding author, upon reasonable request.

## Supplementary Materials

The following supporting information can be downloaded at: https://www.mdpi.com/article/10.3390/vaccines10071009/s1, Figure S1. Number of registered COVID-19 infections and COVID-19 caused deaths in Hungary according to the European Centre for Disease Prevention and Control reports. Table S1. Sociodemographic and clinical characteristics of adults at 1 April 2021 in Hungary by vaccination status registered in the period of 1 April 2021–20 June 2021. Table S2. Distribution of adults according to their general medical practice (GMP) characteristics by vaccination status in the period of 1 April 2021–20 June 2021. Table S3. Sociodemographic and clinical characteristics of adults at 21 June 2021 in Hungary by vaccination status registered in the period of 21 June 2021–15 August 2021. Table S4. Distribution of adults of vaccination cohorts by their general medical practice (GMP) characteristics in the period of 21 June 2021–15 August 2021. Table S5 Application of different vaccines among patients with chronic diseases. Table S6. Mortality risk reduction among vaccinated adults by Cox proportional hazards regression models between 1 April 2021 and 20 June 2021 (adjusted hazard ratios with corresponding 95% confidence intervals, significant results in bold) in Hungary. Table S7. Mortality risk reduction among vaccinated adults by Cox proportional hazards regression models between 21 June 2021 and 15 August 2021 (adjusted hazard ratios with corresponding 95% confidence intervals, significant results in bold) in Hungary.
