# Supplementary material for: Effectiveness of COVID-19 Vaccination in Preventing All-Cause Mortality among Adults during the Third Wave of the Epidemic in Hungary: Nationwide Retrospective Cohort Study
Source: Vaccines (Basel). 2022 Jun 24;10(7):1009. doi: 10.3390/vaccines10071009 (PMC9319484; doi:10.3390/vaccines10071009)
Supplement: Supplementary file 1 [file vaccines-10-01009-s001.zip › vaccines-1748835-supplementary.pdf]

Supplementary Materials

Effectiveness of COVID-19 Vaccination in Preventing All-Cause Mortality among Adults during the Third Wave of the Epidemic in Hungary: Nationwide Retrospective Cohort Study

Anita Pálinkás[1,*] and János Sándor[1]

Figure S1. Number of registered COVID-19 infections and COVID-19 caused deaths in Hungary according to the European Centre for Disease Prevention and Control reports.

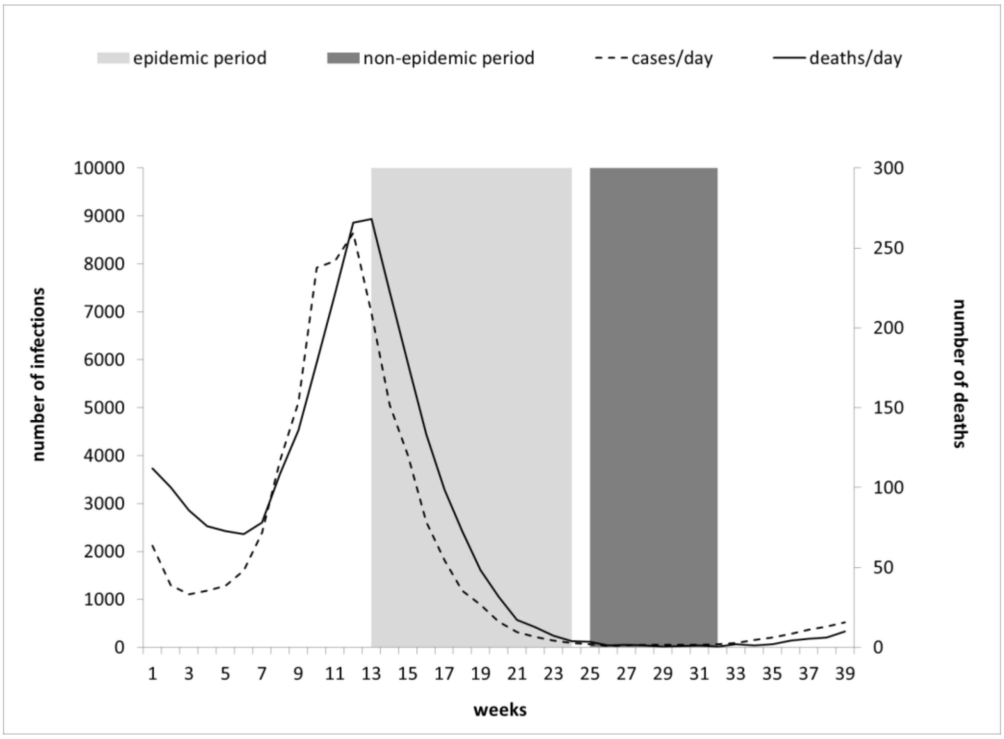

Table S1. Sociodemographic and clinical characteristics of adults at 1 April 2021 in Hungary by vaccination status registered in the period of 1 April 2021–20 June 2021

		**AstraZeneca**	**Janssen**	**Moderna**	**Pfizer**	**Sinopharm**	**Sputnik**	**p-value ***	**Fully vaccinated**	**Not vaccinated**	**p-value ****	**Total**
Age in years, mean (±SD)		56.4 (± 13.8)	45.0 (± 15.7)	56.9 (± 18.3)	57.9 (± 18.3)	58.2 (± 18.4)	50.1 (± 14.7)	< 0.001	55.9 (± 17.6)	45.6 (± 16.6)	< 0.001	55.9 (± 17.6)
Sex, N (%)	male	165325 (49.6%)	46205 (58.9%)	100483 (41.3%)	596890 (37.2%)	443622 (48.2%)	454599 (53.6%)	< 0.001	1807124 (44.9%)	1170758 (49.2%)	< 0.001	2977882 (46.5%)
	female	168155 (50.4%)	32190 (41.1%)	142933 (58.7%)	1007360 (62.8%)	475988 (51.8%)	393099 (46.4%)		2219725 (55.1%)	1207095 (50.8%)		3426820 (53.5%)
Exemption certificate, N (%)	no	321200 (96.3%)	76466 (97.5%)	234819 (96.5%)	1542524 (96.2%)	889769 (96.8%)	837386 (98.8%)	< 0.001	3902164 (96.9%)	2329692 (98.0%)	< 0.001	6231856 (97.3%)
	yes	12280 (3.7%)	1929 (2.5%)	8597 (3.5%)	61726 (3.8%)	29841 (3.2%)	10312 (1.2%)		124685 (3.1%)	48161 (2.0%)		172846 (2.7%)
Diabetes mellitus, N (%)	no	272960 (81.9%)	74787 (95.4%)	217545 (89.4%)	1425390 (88.9%)	812699 (88.4%)	803827 (94.8%)	< 0.001	3607208 (89.6%)	2278045 (95.8%)	< 0.001	5885253 (91.9%)
	yes	60520 (18.1%)	3608 (4.6%)	25871 (10.6%)	178860 (11.1%)	106911 (11.6%)	43871 (5.2%)		419641 (10.4%)	99808 (4.2%)		519449 (8.1%)
Ischemic heart disease, N (%)	no	317706 (95.3%)	77358 (98.7%)	233734 (96.0%)	1534513 (95.7%)	884248 (96.2%)	835639 (98.6%)	< 0.001	3883198 (96.4%)	2341062 (98.5%)	< 0.001	6224260 (97.2%)
	yes	15774 (4.7%)	1037 (1.3%)	9682 (4.0%)	69737 (4.3%)	35362 (3.8%)	12059 (1.4%)		143651 (3.6%)	36791 (1.5%)		180442 (2.8%)
COPD, N (%)	no	319425 (95.8%)	77060 (98.3%)	234780 (96.5%)	1544896 (96.3%)	886993 (96.5%)	834795 (98.5%)	< 0.001	3897949 (96.8%)	2337354 (98.3%)	< 0.001	6235303 (97.4%)
	yes	14055 (4.2%)	1335 (1.7%)	8636 (3.5%)	59354 (3.7%)	32617 (3.5%)	12903 (1.5%)		128900 (3.2%)	40499 (1.7%)		169399 (2.6%)
Asthma, N (%)	no	333417 (99.98%)	78390 (99.99%)	243394 (99.99%)	1604076 (99.99%)	919546 (99.99%)	847658 (99.995%)	< 0.001	4026481 (99.99%)	2377685 (99.99%)	0.006	6404166 (99.99%)
	yes	63 (0.02%)	5 (0.01%)	22 (0.01%)	174 (0.01%)	64 (0.01%)	40 (0.005%)		368 (0.01%)	168 (0.01%)		536 (0.01%)
Hypertension, N (%)	no	154980 (46.5%)	61114 (78.0%)	134964 (55.4%)	854979 (53.3%)	466203 (50.7%)	585604 (69.1%)	< 0.001	2257844 (56.1%)	1892447 (79.6%)	< 0.001	4150291 (64.8%)
	yes	178500 (53.5%)	17281 (22.0%)	108452 (44.6%)	749271 (46.7%)	453407 (49.3%)	262094 (30.9%)		1769005 (43.9%)	485406 (20.4%)		2254411 (35.2%)
Cancer, N (%)	no	314763 (94.4%)	77602 (99.0%)	231834 (95.2%)	1517819 (94.6%)	887926 (96.6%)	839655 (99.1%)	< 0.001	3869599 (96.1%)	2342679 (98.5%)	< 0.001	6212278 (97.0%)
	yes	18717 (5.6%)	793 (1.0%)	11582 (4.8%)	86431 (5.4%)	31684 (3.4%)	8043 (0.9%)		157250 (3.9%)	35174 (1.5%)		192424 (3.0%)
Chronic liver disease, N (%)	no	333327 (99.95%)	78367 (99.96%)	243308 (99.96%)	1603773 (99.97%)	919399 (99.98%)	847622 (99.99%)	< 0.001	4025796 (99.97%)	2377202 (99.97%)	0.357	6402998 (99.97%)
	yes	153 (0.05%)	28 (0.04%)	108 (0.04%)	477 (0.03%)	211 (0.02%)	76 (0.01%)		1053 (0.03%)	651 (0.03%)		1704 (0.03%)
End-stage renal disease, N (%)	no	332490 (99.7%)	78357 (99.95%)	242725 (99.7%)	1599375 (99.7%)	918529 (99.9%)	847581 (99.99%)	< 0.001	4019057 (99.8%)	2375987 (99.9%)	< 0.001	6395044 (99.8%)
	yes	990 (0.3%)	38 (0.05%)	691 (0.3%)	4875 (0.3%)	1081 (0.1%)	117 (0.01%)		7792 (0.2%)	1866 (0.1%)		9658 (0.2%)
Total, N (%)		333480 (5.2%)	78395 (1.2%)	243416 (3.8%)	1604250 (25.0%)	919610 (14.4%)	847698 (13.2%)	-	4026849 (62.9%)	2377853 (37.1%)	-	6404702 (100%)

* t-test/ANOVA/χ^2-test by type of vaccine

** t-test/ANOVA/χ^2-test comparing vaccinated and not vaccinated subjects

Table S2. Distribution of adults according to their general medical practice (GMP) characteristics by vaccination status in the period of 1 April 2021–20 June 2021

		AstraZeneca	Janssen	Moderna	Pfizer	Sinopharm	Sputnik	p-value*	Fully vaccinated	Not vaccinated	p-value**	Total
County of GMP, N (%)	Budapest	68371 (20.5%)	14792 (18.9%)	69317 (28.5%)	329575 (20.5%)	179812 (19.6%)	180328 (21.3%)	< 0.001	842195 (20.9%)	314369 (13.2%)	< 0.001	1156564 (18.1%)
	Baranya	13223 (4.0%)	2639 (3.4%)	7879 (3.2%)	68285 (4.3%)	35189 (3.8%)	30519 (3.6%)		157734 (3.9%)	88423 (3.7%)		246157 (3.8%)
	Bács-Kiskun	16286 (4.9%)	4273 (5.5%)	10449 (4.3%)	79188 (4.9%)	46257 (5%)	39222 (4.6%)		195675 (4.9%)	143250 (6.0%)		338925 (5.3%)
	Békés	8666 (2.6%)	1531 (2.0%)	5789 (2.4%)	54379 (3.4%)	28383 (3.1%)	26146 (3.1%)		124894 (3.1%)	94002 (4.0%)		218896 (3.4%)
	Borsod-Abaúj-Zemplén	17175 (5.2%)	7403 (9.4%)	12540 (5.2%)	81254 (5.1%)	55065 (6.0%)	51697 (6.1%)		225134 (5.6%)	190256 (8.0%)		415390 (6.5%)
	Csongrád-Csanád	12617 (3.8%)	4066 (5.2%)	7798 (3.2%)	67715 (4.2%)	39672 (4.3%)	32543 (3.8%)		164411 (4.1%)	99472 (4.2%)		263883 (4.1%)
	Fejér	16262 (4.9%)	3530 (4.5%)	9525 (3.9%)	70185 (4.4%)	42395 (4.6%)	32494 (3.8%)		174391 (4.3%)	100159 (4.2%)		274550 (4.3%)
	Győr-Moson-Sopron	16391 (4.9%)	3726 (4.8%)	10182 (4.2%)	76234 (4.8%)	43196 (4.7%)	43791 (5.2%)		193520 (4.8%)	102212 (4.3%)		295732 (4.6%)
	Hajdú-Bihar	16798 (5%)	3257 (4.2%)	10794 (4.4%)	78593 (4.9%)	44107 (4.8%)	39765 (4.7%)		193314 (4.8%)	150225 (6.3%)		343539 (5.4%)
	Heves	9080 (2.7%)	1864 (2.4%)	6116 (2.5%)	47827 (3.0%)	27633 (3.0%)	23421 (2.8%)		115941 (2.9%)	79651 (3.3%)		195592 (3.1%)
	Komárom-Esztergom	9140 (2.7%)	2130 (2.7%)	8382 (3.4%)	44685 (2.8%)	27864 (3.0%)	29997 (3.5%)		122198 (3.0%)	80396 (3.4%)		202594 (3.2%)
	Nógrád	7032 (2.1%)	2154 (2.7%)	3185 (1.3%)	32028 (2.0%)	16287 (1.8%)	10197 (1.2%)		70883 (1.8%)	54669 (2.3%)		125552 (2.0%)
	Pest	48356 (14.5%)	12500 (15.9%)	39850 (16.4%)	194110 (12.1%)	126768 (13.8%)	113557 (13.4%)		535141 (13.3%)	264386 (11.1%)		799527 (12.5%)
	Somogy	10863 (3.3%)	1688 (2.2%)	5640 (2.3%)	49459 (3.1%)	27937 (3.0%)	26727 (3.2%)		122314 (3.0%)	77068 (3.2%)		199382 (3.1%)
	Szabolcs-Szatmár-Bereg	17880 (5.4%)	3917 (5.0%)	7728 (3.2%)	81965 (5.1%)	47645 (5.2%)	43554 (5.1%)		202689 (5.0%)	151266 (6.4%)		353955 (5.5%)
	Jász-Nagykun-Szolnok	10722 (3.2%)	2467 (3.1%)	6361 (2.6%)	54806 (3.4%)	31787 (3.5%)	27160 (3.2%)		133303 (3.3%)	117277 (4.9%)		250580 (3.9%)
	Tolna	7398 (2.2%)	1285 (1.6%)	4571 (1.9%)	40613 (2.5%)	20272 (2.2%)	17810 (2.1%)		91949 (2.3%)	53942 (2.3%)		145891 (2.3%)
	Vas	7965 (2.4%)	1112 (1.4%)	5098 (2.1%)	45892 (2.9%)	24579 (2.7%)	22187 (2.6%)		106833 (2.7%)	60971 (2.6%)		167804 (2.6%)
	Veszprém	10146 (3%)	2356 (3.0%)	6856 (2.8%)	59174 (3.7%)	29308 (3.2%)	31907 (3.8%)		139747 (3.5%)	88677 (3.7%)		228424 (3.6%)
	Zala	9109 (2.7%)	1705 (2.2%)	5356 (2.2%)	48283 (3.0%)	25454 (2.8%)	24676 (2.9%)		114583 (2.8%)	67182 (2.8%)		181765 (2.8%)
Size of GMP, N (%)	≤ 800 patients	6226 (1.9%)	1705 (2.2%)	4913 (2.0%)	38440 (2.4%)	21129 (2.3%)	16449 (1.9%)	< 0.001	88862 (2.2%)	57006 (2.4%)	< 0.001	145868 (2.3%)
	801-1200 patients	33905 (10.2%)	8990 (11.5%)	23632 (9.7%)	181525 (11.3%)	111371 (12.1%)	87804 (10.4%)		447227 (11.1%)	291574 (12.3%)		738801 (11.5%)
	1201-1600 patients	95726 (28.7%)	23319 (29.7%)	67455 (27.7%)	468837 (29.2%)	271817 (29.6%)	247325 (29.2%)		1174479 (29.2%)	713646 (30.0%)		1888125 (29.5%)
	1601-2000 patients	101766 (30.5%)	23485 (30.0%)	76553 (31.4%)	481614 (30.0%)	273011 (29.7%)	257969 (30.4%)		1214398 (30.2%)	702408 (29.5%)		1916806 (29.9%)
	> 2000 patients	95857 (28.7%)	20896 (26.7%)	70863 (29.1%)	433834 (27.0%)	242282 (26.3%)	238151 (28.1%)		1101883 (27.4%)	613219 (25.8%)		1715102 (26.8%)
Settlement type of GMP, N (%)	rural	79897 (24.0%)	27431 (35.0%)	52005 (21.4%)	388090 (24.2%)	252014 (27.4%)	206877 (24.4%)	< 0.001	1006314 (25.0%)	750438 (31.6%)	< 0.001	1756752 (27.4%)
	urban	253583 (76.0%)	50964 (65.0%)	191411 (78.6%)	1216160 (75.8%)	667596 (72.6%)	640821 (75.6%)		3020535 (75.0%)	1627415 (68.4%)		4647950 (72.6%)
Age and sex of GP, N (%)	< 65 years male	102389 (30.7%)	24050 (30.7%)	72633 (29.8%)	479111 (29.9%)	277257 (30.1%)	256132 (30.2%)	< 0.001	1211572 (30.1%)	740118 (31.1%)	< 0.001	1951690 (30.5%)
	< 65 years female	113511 (34.0%)	24472 (31.2%)	88363 (36.3%)	545213 (34.0%)	298775 (32.5%)	291999 (34.4%)		1362333 (33.8%)	742381 (31.2%)		2104714 (32.9%)
	≥ 65 years male	53426 (16.0%)	13227 (16.9%)	36292 (14.9%)	253513 (15.8%)	155240 (16.9%)	133380 (15.7%)		645078 (16.0%)	399494 (16.8%)		1044572 (16.3%)
	≥ 65 years female	47724 (14.3%)	9933 (12.7%)	35393 (14.5%)	234420 (14.6%)	130406 (14.2%)	120627 (14.2%)		578503 (14.4%)	299578 (12.6%)		878081 (13.7%)
	vacant	16430 (4.9%)	6713 (8.6%)	10735 (4.4%)	91993 (5.7%)	57932 (6.3%)	45560 (5.4%)		229363 (5.7%)	196282 (8.3%)		425645 (6.6%)
Relative education for GMP, mean (±SD)		1.007 (± 0.077)	0.982 (± 0.089)	1.017 (± 0.078)	1.006 (± 0.079)	0.999 (± 0.081)	1.007 (± 0.077)	< 0.001	1.005 (± 0.079)	0.979 (± 0.082)	< 0.001	1.005 (± 0.079)
Relative employment for GMP, mean (±SD)		1.010 (± 0.100)	0.979 (± 0.132)	1.022 (± 0.095)	1.007 (± 0.103)	1.001 (± 0.108)	1.011 (± 0.100)	< 0.001	1.007 (± 0.103)	0.976 (± 0.120)	< 0.001	1.007 (± 0.103)
Total. N (%)		333480 (5.2%)	78395 (1.2%)	243416 (3.8%)	1604250 (25.0%)	919610 (14.4%)	847698 (13.2%)	-	4026849 (62.9%)	2377853 (37.1%)	-	6404702 (100%)

* t-test/ANOVA/χ^2-test by type of vaccine ** t-test/ANOVA/χ^2-test comparing vaccinated and not vaccinated subjects

Table S3. Sociodemographic and clinical characteristics of adults at 21 June 2021 in Hungary by vaccination status registered in the period of 21 June 2021–15 August 2021

		AstraZeneca	Janssen	Moderna	Pfizer	Sinopharm	Sputnik	p-value*	Fully vaccinated	Not vaccinated	p-value**	Total
Age in years, mean (±SD)		53.0 (± 14.9)	44.6 (± 15.6)	54.5 (± 18.3)	53.8 (± 18.9)	57.3 (± 18.6)	49.9 (± 14.8)	< 0.001	53.6 (± 17.8)	45.5 (± 16.5)	< 0.001	51.1 (± 17.8)
Sex, N (%)	male	311473 (52.5%)	57560 (58.4%)	136173 (42.3%)	840773 (39.5%)	474909 (48.5%)	465633 (53.7%)	< 0.001	2286521 (45.8%)	1098801 (49.4%)	< 0.001	3385322 (46.9%)
	female	281928 (47.5%)	40994 (41.6%)	185802 (57.7%)	1288669 (60.5%)	504533 (51.5%)	401164 (46.3%)		2703090 (54.2%)	1127378 (50.6%)		3830468 (53.1%)
Exemption certificate, N (%)	no	575425 (97.0%)	96194 (97.6%)	311222 (96.7%)	2059265 (96.7%)	948327 (96.8%)	856332 (98.8%)	< 0.001	4846765 (97.1%)	2182314 (98.0%)	< 0.001	7029079 (97.4%)
	yes	17976 (3.0%)	2360 (2.4%)	10753 (3.3%)	70177 (3.3%)	31115 (3.2%)	10465 (1.2%)		142846 (2.9%)	43865 (2.0%)		186711 (2.6%)
Diabetes mellitus, N (%)	no	515935 (86.9%)	94232 (95.6%)	290920 (90.4%)	1929423 (90.6%)	870651 (88.9%)	822541 (94.9%)	< 0.001	4523702 (90.7%)	2136134 (96.0%)	< 0.001	6659836 (92.3%)
	yes	77466 (13.1%)	4322 (4.4%)	31055 (9.6%)	200019 (9.4%)	108791 (11.1%)	44256 (5.1%)		465909 (9.3%)	90045 (4.0%)		555954 (7.7%)
Ischemic heart disease, N (%)	no	573125 (96.6%)	97297 (98.7%)	310635 (96.5%)	2053804 (96.4%)	943542 (96.3%)	854646 (98.6%)	< 0.001	4833049 (96.9%)	2193181 (98.5%)	< 0.001	7026230 (97.4%)
	yes	20276 (3.4%)	1257 (1.3%)	11340 (3.5%)	75638 (3.6%)	35900 (3.7%)	12151 (1.4%)		156562 (3.1%)	32998 (1.5%)		189560 (2.6%)
COPD, N (%)	no	574127 (96.8%)	96905 (98.3%)	311517 (96.8%)	2062925 (96.9%)	946011 (96.6%)	853782 (98.5%)	< 0.001	4845267 (97.1%)	2189777 (98.4%)	< 0.001	7035044 (97.5%)
	yes	19274 (3.2%)	1649 (1.7%)	10458 (3.2%)	66517 (3.1%)	33431 (3.4%)	13015 (1.5%)		144344 (2.9%)	36402 (1.6%)		180746 (2.5%)
Asthma, N (%)	no	593319 (99.99%)	98549 (99.99%)	321944 (99.99%)	2129217 (99.99%)	979374 (99.99%)	866755 (99.995%)	< 0.001	4989158 (99.99%)	2226045 (99.99%)	< 0.001	7215203 (99.99%)
	yes	82 (0.01%)	5 (0.01%)	31 (0.01%)	225 (0.01%)	68 (0.01%)	42 (0.005%)		453 (0.01%)	134 (0.01%)		587 (0.01%)
Hypertension, N (%)	no	336984 (56.8%)	77802 (78.9%)	191286 (59.4%)	1281562 (60.2%)	515006 (52.6%)	602182 (69.5%)	< 0.001	3004822 (60.2%)	1782685 (80.1%)	< 0.001	4787507 (66.3%)
	yes	256417 (43.2%)	20752 (21.1%)	130689 (40.6%)	847880 (39.8%)	464436 (47.4%)	264615 (30.5%)		1984789 (39.8%)	443494 (19.9%)		2428283 (33.7%)
Cancer, N (%)	no	570528 (96.1%)	97591 (99.0%)	308552 (95.8%)	2034115 (95.5%)	947378 (96.7%)	858680 (99.1%)	< 0.001	4816844 (96.5%)	2196777 (98.7%)	< 0.001	7013621 (97.2%)
	yes	22873 (3.9%)	963 (1.0%)	13423 (4.2%)	95327 (4.5%)	32064 (3.3%)	8117 (0.9%)		172767 (3.5%)	29402 (1.3%)		202169 (2.8%)
Chronic liver disease, N (%)	no	593185 (99.96%)	98515 (99.96%)	321840 (99.96%)	2128846 (99.97%)	979221 (99.98%)	866718 (99.99%)	< 0.001	4988325 (99.97%)	2225695 (99.98%)	0.001	7214020 (99.98%)
	yes	216 (0.04%)	39 (0.04%)	135 (0.04%)	596 (0.03%)	221 (0.02%)	79 (0.01%)		1286 (0.03%)	484 (0.02%)		1770 (0.02%)
End-stage renal disease, N (%)	no	592225 (99.8%)	98509 (99.95%)	321175 (99.8%)	2124155 (99.8%)	978360 (99.9%)	866676 (99.99%)	< 0.001	4981100 (99.8%)	2224754 (99.9%)	< 0.001	7205854 (99.9%)
	yes	1176 (0.2%)	45 (0.05%)	800 (0.2%)	5287 (0.2%)	1082 (0.1%)	121 (0.01%)		8511 (0.2%)	1425 (0.1%)		9936 (0.1%)
Total, N (%)		593401 (8.2%)	98554 (1.4%)	321975 (4.5%)	2129442 (29.5%)	979442 (13.6%)	866797 (12%)	-	4989611 (69.1%)	2226179 (30.9%)	-	7215790 (100%)

* t-test/ANOVA/χ^2-test by type of vaccine ** t-test/ANOVA/χ^2-test comparing vaccinated and not vaccinated subjects

Table S4. Distribution of adults of vaccination cohorts by their general medical practice (GMP) characteristics in the period of 21 June 2021–15 August 2021

		AstraZeneca	Janssen	Moderna	Pfizer	Sinopharm	Sputnik	p-value*	Fully vaccinated	Not vaccinated	p-value**	Total
County of GMP, N (%)	Budapest	130173 (21.9%)	18310 (18.6%)	90643 (28.2%)	436521 (20.5%)	190139 (19.4%)	183106 (21.1%)	< 0.001	1048892 (21%)	289766 (13.0%)	< 0.001	1338658 (18.6%)
	Baranya	22211 (3.7%)	3263 (3.3%)	9674 (3.0%)	84181 (4.0%)	37256 (3.8%)	31023 (3.6%)		187608 (3.8%)	83180 (3.7%)		270788 (3.8%)
	Bács-Kiskun	28465 (4.8%)	5404 (5.5%)	14693 (4.6%)	99804 (4.7%)	49025 (5.0%)	40314 (4.7%)		237705 (4.8%)	135466 (6.1%)		373171 (5.2%)
	Békés	18405 (3.1%)	2335 (2.4%)	6685 (2.1%)	73658 (3.5%)	29792 (3.0%)	26628 (3.1%)		157503 (3.2%)	89013 (4%)		246516 (3.4%)
	Borsod-Abaúj-Zemplén	36390 (6.1%)	9454 (9.6%)	17197 (5.3%)	111992 (5.3%)	58624 (6.0%)	53197 (6.1%)		286854 (5.7%)	179409 (8.1%)		466263 (6.5%)
	Csongrád-Csanád	23899 (4.0%)	4897 (5%)	10337 (3.2%)	89774 (4.2%)	41572 (4.2%)	33267 (3.8%)		203746 (4.1%)	93082 (4.2%)		296828 (4.1%)
	Fejér	26802 (4.5%)	4340 (4.4%)	12546 (3.9%)	94968 (4.5%)	45146 (4.6%)	33671 (3.9%)		217473 (4.4%)	93248 (4.2%)		310721 (4.3%)
	Győr-Moson-Sopron	31726 (5.3%)	4915 (5.0%)	13643 (4.2%)	106469 (5.0%)	45575 (4.7%)	44685 (5.2%)		247013 (5%)	93835 (4.2%)		340848 (4.7%)
	Hajdú-Bihar	27289 (4.6%)	3895 (4.0%)	14205 (4.4%)	104841 (4.9%)	46877 (4.8%)	41056 (4.7%)		238163 (4.8%)	142104 (6.4%)		380267 (5.3%)
	Heves	16273 (2.7%)	2261 (2.3%)	7043 (2.2%)	65685 (3.1%)	29407 (3.0%)	23859 (2.8%)		144528 (2.9%)	74902 (3.4%)		219430 (3%)
	Komárom-Esztergom	18195 (3.1%)	2896 (2.9%)	10815 (3.4%)	62858 (3.0%)	29926 (3.1%)	30750 (3.5%)		155440 (3.1%)	74984 (3.4%)		230424 (3.2%)
	Nógrád	11375 (1.9%)	2572 (2.6%)	4302 (1.3%)	41940 (2.0%)	18290 (1.9%)	10557 (1.2%)		89036 (1.8%)	50919 (2.3%)		139955 (1.9%)
	Pest	78207 (13.2%)	15512 (15.7%)	53085 (16.5%)	255361 (12.0%)	135285 (13.8%)	116670 (13.5%)		654120 (13.1%)	246278 (11.1%)		900398 (12.5%)
	Somogy	14826 (2.5%)	2232 (2.3%)	7487 (2.3%)	64828 (3.0%)	29431 (3.0%)	27261 (3.1%)		146065 (2.9%)	71896 (3.2%)		217961 (3.0%)
	Szabolcs-Szatmár-Bereg	27184 (4.6%)	5033 (5.1%)	11921 (3.7%)	112669 (5.3%)	52972 (5.4%)	44630 (5.1%)		254409 (5.1%)	140911 (6.3%)		395320 (5.5%)
	Jász-Nagykun-Szolnok	16694 (2.8%)	3074 (3.1%)	9124 (2.8%)	71851 (3.4%)	35169 (3.6%)	27825 (3.2%)		163737 (3.3%)	111141 (5.0%)		274878 (3.8%)
	Tolna	11497 (1.9%)	1857 (1.9%)	5522 (1.7%)	52947 (2.5%)	21545 (2.2%)	18141 (2.1%)		111509 (2.2%)	51359 (2.3%)		162868 (2.3%)
	Vas	16075 (2.7%)	1359 (1.4%)	6809 (2.1%)	61192 (2.9%)	26075 (2.7%)	22587 (2.6%)		134097 (2.7%)	57394 (2.6%)		191491 (2.7%)
	Veszprém	21206 (3.6%)	2950 (3.0%)	9237 (2.9%)	76267 (3.6%)	30518 (3.1%)	32570 (3.8%)		172748 (3.5%)	83847 (3.8%)		256595 (3.6%)
	Zala	16509 (2.8%)	1995 (2.0%)	7007 (2.2%)	61636 (2.9%)	26818 (2.7%)	25000 (2.9%)		138965 (2.8%)	63445 (2.8%)		202410 (2.8%)
Size of GMP, N (%)	≤ 800 patients	10652 (1.8%)	2186 (2.2%)	6288 (2.0%)	48797 (2.3%)	22588 (2.3%)	16866 (1.9%)	< 0.001	107377 (2.2%)	53467 (2.4%)	< 0.001	160844 (2.2%)
	801-1200 patients	58711 (9.9%)	11260 (11.4%)	31045 (9.6%)	237267 (11.1%)	118862 (12.1%)	89853 (10.4%)		546998 (11%)	274038 (12.3%)		821036 (11.4%)
	1201-1600 patients	169218 (28.5%)	29327 (29.8%)	89659 (27.8%)	620499 (29.1%)	289175 (29.5%)	252599 (29.1%)		1450477 (29.1%)	669339 (30.1%)		2119816 (29.4%)
	1601-2000 patients	182838 (30.8%)	29346 (29.8%)	100788 (31.3%)	640859 (30.1%)	290401 (29.6%)	263658 (30.4%)		1507890 (30.2%)	656987 (29.5%)		2164877 (30.0%)
	> 2000 patients	171982 (29.0%)	26435 (26.8%)	94195 (29.3%)	582020 (27.3%)	258416 (26.4%)	243821 (28.1%)		1376869 (27.6%)	572348 (25.7%)		1949217 (27.0%)
Settlement type of GMP, N (%)	rural	140995 (23.8%)	34232 (34.7%)	72814 (22.6%)	524822 (24.6%)	272299 (27.8%)	212680 (24.5%)	< 0.001	1257842 (25.2%)	704027 (31.6%)	< 0.001	1961869 (27.2%)
	urban	452406 (76.2%)	64322 (65.3%)	249161 (77.4%)	1604620 (75.4%)	707143 (72.2%)	654117 (75.5%)		3731769 (74.8%)	1522152 (68.4%)		5253921 (72.8%)
Age and sex of GP, N (%)	< 65 years male	184107 (31.0%)	30396 (30.8%)	95885 (29.8%)	639075 (30.0%)	296075 (30.2%)	262426 (30.3%)	< 0.001	1507964 (30.2%)	693791 (31.2%)	< 0.001	2201755 (30.5%)
	< 65 years female	203694 (34.3%)	30664 (31.1%)	115206 (35.8%)	721676 (33.9%)	317205 (32.4%)	298047 (34.4%)		1686492 (33.8%)	693632 (31.2%)		2380124 (33.0%)
	≥ 65 years male	93374 (15.7%)	16565 (16.8%)	48261 (15.0%)	337331 (15.8%)	165666 (16.9%)	136519 (15.7%)		797716 (16%)	374404 (16.8%)		1172120 (16.2%)
	≥ 65 years female	83174 (14%)	12160 (12.3%)	47075 (14.6%)	307101 (14.4%)	138010 (14.1%)	122929 (14.2%)		710449 (14.2%)	279432 (12.6%)		989881 (13.7%)
	vacant	29052 (4.9%)	8769 (8.9%)	15548 (4.8%)	124259 (5.8%)	62486 (6.4%)	46876 (5.4%)		286990 (5.8%)	184920 (8.3%)		471910 (6.5%)
Relative education for GMP, mean (±SD)		1.009 (± 0.077)	0.981 (± 0.089)	1.014 (± 0.080)	1.004 (± 0.079)	0.998 (± 0.081)	1.006 (± 0.077)	< 0.001	1.004 (± 0.079)	0.978 (± 0.082)	< 0.001	0.996 (± 0.081)
Relative employment for GMP, mean (±SD)		1.013 (± 0.099)	0.977 (± 0.134)	1.018 (± 0.099)	1.006 (± 0.104)	0.999 (± 0.110)	1.011 (± 0.100)	< 0.001	1.007 (± 0.104)	0.975 (± 0.120)	< 0.001	0.997 (± 0.110)
Total, N (%)		593401 (8.2%)	98554 (1.4%)	321975 (4.5%)	2129442 (29.5%)	979442 (13.6%)	866797 (12%)	-	4989611 (69.1%)	2226179 (30.9%)	-	7215790 (100%)

* t-test/ANOVA/χ^2-test by type of vaccine ** t-test/ANOVA/χ^2-test comparing vaccinated and not vaccinated subjects

Table S5 Application of different vaccines among patients with chronic diseases.

		**AstraZeneca**	**Janssen**	**Moderna**	**Pfizer**	**Sinopharm**	**Sputnik**	**None**	**Total**
Number of patients in epidemic period (%)	Diabetes mellitus	60520 (11.7%)	3608 (0.7%)	25871 (5.0%)	178860 (34.4%)	106911 (20.6%)	43871 (8.4%)	99808 (19.2%)	519449 (100%)
	Ischemic heart disease	15774 (8.7%)	1037 (0.6%)	9682 (5.4%)	69737 (38.6%)	35362 (19.6%)	12059 (6.7%)	36791 (20.4%)	180442 (100%)
	COPD	14055 (8.3%)	1335 (0.8%)	8636 (5.1%)	59354 (35.0%)	32617 (19.3%)	12903 (7.6%)	40499 (23.9%)	169399 (100%)
	Asthma	63 (11.8%)	5 (0.9%)	22 (4.1%)	174 (32.5%)	64 (11.9%)	40 (7.5%)	168 (31.3%)	536 (100%)
	Hypertension	178500 (7.9%)	17281 (0.8%)	108452 (4.8%)	749271 (33.2%)	453407 (20.1%)	262094 (11.6%)	485406 (21.5%)	2254411 (100%)
	Cancer	18717 (9.7%)	793 (0.4%)	11582 (6.0%)	86431 (44.9%)	31684 (16.5%)	8043 (4.2%)	35174 (18.3%)	192424 (100%)
	Chronic liver disease	153 (9.0%)	28 (1.6%)	108 (6.3%)	477 (28.0%)	211 (12.4%)	76 (4.5%)	651 (38.2%)	1704 (100%)
	End-stage renal disease	990 (10.3%)	38 (0.4%)	691 (7.2%)	4875 (50.5%)	1081 (11.2%)	117 (1.2%)	1866 (19.3%)	9658 (100%)
Number of patients in non-epidemic period (%)	Diabetes mellitus	77466 (13.9%)	4322 (0.8%)	31055 (5.6%)	200019 (36.0%)	108791 (19.6%)	44256 (8.0%)	90045 (16.2%)	555954 (100%)
	Ischemic heart disease	20276 (10.7%)	1257 (0.7%)	11340 (6%)	75638 (39.9%)	35900 (18.9%)	12151 (6.4%)	32998 (17.4%)	189560 (100%)
	COPD	19274 (10.7%)	1649 (0.9%)	10458 (5.8%)	66517 (36.8%)	33431 (18.5%)	13015 (7.2%)	36402 (20.1%)	180746 (100%)
	Asthma	82 (14%)	5 (0.9%)	31 (5.3%)	225 (38.3%)	68 (11.6%)	42 (7.2%)	134 (22.8%)	587 (100%)
	Hypertension	256417 (10.6%)	20752 (0.9%)	130689 (5.4%)	847880 (34.9%)	464436 (19.1%)	264615 (10.9%)	443494 (18.3%)	2428283 (100%)
	Cancer	22873 (11.3%)	963 (0.5%)	13423 (6.6%)	95327 (47.2%)	32064 (15.9%)	8117 (4.0%)	29402 (14.5%)	202169 (100%)
	Chronic liver disease	216 (12.2%)	39 (2.2%)	135 (7.6%)	596 (33.7%)	221 (12.5%)	79 (4.5%)	484 (27.3%)	1770 (100%)
	End-stage renal disease	1176 (11.8%)	45 (0.5%)	800 (8.1%)	5287 (53.2%)	1082 (10.9%)	121 (1.2%)	1425 (14.3%)	9936 (100%)

Table S6. Mortality risk reduction among vaccinated adults by Cox proportional hazards regression models between 1 April 2021 and 20 June 2021 (adjusted hazard ratios with corresponding 95% confidence intervals, significant results in bold) in Hungary

		**AstraZeneca**	**Janssen**	**Moderna**	**Pfizer**	**Sinopharm**	**Sputnik**
Vaccination	Fully / Not vaccinated	**0.129 [0.111 - 0.150]**	**0.174 [0.118 - 0.255]**	**0.187 [0.173 - 0.202]**	**0.197 [0.190 - 0.203]**	**0.147 [0.140 - 0.154]**	**0.098 [0.088 - 0.108]**
Age in years		**1.089 [1.088 - 1.090]**	**1.089 [1.088 - 1.091]**	**1.090 [1.089 - 1.091]**	**1.091 [1.090 - 1.092]**	**1.091 [1.090 - 1.092]**	**1.090 [1.089 - 1.091]**
Sex	Female / Male	**0.567 [0.550 - 0.584]**	**0.568 [0.551 - 0.585]**	**0.571 [0.555 - 0.589]**	**0.602 [0.586 - 0.618]**	**0.560 [0.545 - 0.577]**	**0.565 [0.548 - 0.582]**
Exemption certificate	Yes / No	**1.635 [1.547 - 1.726]**	**1.622 [1.535 - 1.714]**	**1.615 [1.530 - 1.705]**	**1.656 [1.578 - 1.737]**	**1.591 [1.509 - 1.677]**	**1.622 [1.535 - 1.713]**
Diabetes mellitus	Yes / No	**1.423 [1.369 - 1.480]**	**1.424 [1.370 - 1.481]**	**1.420 [1.367 - 1.476]**	**1.387 [1.341 - 1.435]**	**1.440 [1.389 - 1.493]**	**1.426 [1.372 - 1.481]**
Ischemic heart disease	Yes / No	**1.466 [1.394 - 1.542]**	**1.464 [1.391 - 1.540]**	**1.457 [1.386 - 1.531]**	**1.420 [1.359 - 1.484]**	**1.477 [1.409 - 1.549]**	**1.478 [1.405 - 1.554]**
COPD	Yes / No	**1.677 [1.593 - 1.766]**	**1.672 [1.588 - 1.762]**	**1.677 [1.594 - 1.765]**	**1.681 [1.607 - 1.759]**	**1.714 [1.632 - 1.800]**	**1.679 [1.595 - 1.768]**
Asthma	Yes / No	**3.418 [2.020 - 5.784]**	**3.423 [2.023 - 5.791]**	**3.268 [1.931 - 5.528]**	**3.077 [1.852 - 5.113]**	**3.706 [2.266 - 6.062]**	**3.416 [2.019 - 5.780]**
Hypertension	Yes / No	**1.341 [1.294 - 1.389]**	**1.340 [1.294 - 1.388]**	**1.331 [1.285 - 1.378]**	**1.255 [1.215 - 1.296]**	**1.302 [1.258 - 1.346]**	**1.336 [1.290 - 1.384]**
Cancer	Yes / No	**4.629 [4.449 - 4.816]**	**4.649 [4.467 - 4.837]**	**4.470 [4.298 - 4.649]**	**3.726 [3.595 - 3.862]**	**4.249 [4.090 - 4.415]**	**4.607 [4.428 - 4.793]**
Chronic liver disease	Yes / No	**13.021 [10.850 - 15.625]**	**12.953 [10.777 - 15.568]**	**13.374 [11.170 - 16.012]**	**12.941 [10.850 - 15.435]**	**12.997 [10.856 - 15.559]**	**13.007 [10.831 - 15.621]**
End-stage renal disease	Yes / No	**4.123 [3.666 - 4.638]**	**4.005 [3.553 - 4.516]**	**3.950 [3.514 - 4.441]**	**3.574 [3.222 - 3.965]**	**4.223 [3.772 - 4.727]**	**3.994 [3.543 - 4.502]**
Location of GMP by county (reference Budapest)	Baranya	0.988 [0.904 - 1.079]	0.991 [0.907 - 1.083]	0.989 [0.906 - 1.079]	1.018 [0.942 - 1.100]	1.009 [0.928 - 1.097]	0.995 [0.911 - 1.086]
	Bács-Kiskun	0.944 [0.870 - 1.024]	0.947 [0.873 - 1.028]	0.952 [0.878 - 1.031]	0.969 [0.901 - 1.043]	0.958 [0.887 - 1.035]	0.952 [0.878 - 1.032]
	Békés	0.958 [0.877 - 1.046]	0.955 [0.874 - 1.044]	0.959 [0.879 - 1.046]	1.023 [0.946 - 1.106]	0.950 [0.873 - 1.034]	0.952 [0.872 - 1.040]
	Borsod-Abaúj-Zemplén	1.019 [0.943 - 1.101]	1.016 [0.940 - 1.098]	1.023 [0.948 - 1.104]	1.064 [0.992 - 1.141]	1.018 [0.945 - 1.096]	1.020 [0.945 - 1.102]
	Csongrád-Csanád	0.933 [0.857 - 1.016]	0.937 [0.861 - 1.020]	0.945 [0.869 - 1.028]	0.984 [0.913 - 1.060]	0.937 [0.865 - 1.016]	0.943 [0.867 - 1.025]
	Fejér	**1.106 [1.015 - 1.204]**	**1.114 [1.023 - 1.214]**	**1.126 [1.036 - 1.224]**	**1.185 [1.099 - 1.277]**	**1.118 [1.031 - 1.213]**	**1.112 [1.021 - 1.210]**
	Győr-Moson-Sopron	1.022 [0.937 - 1.116]	1.022 [0.936 - 1.117]	1.026 [0.941 - 1.119]	1.063 [0.983 - 1.149]	1.023 [0.941 - 1.113]	1.033 [0.947 - 1.127]
	Hajdú-Bihar	**0.880 [0.809 - 0.956]**	**0.883 [0.812 - 0.960]**	**0.889 [0.819 - 0.966]**	**0.917 [0.851 - 0.988]**	**0.869 [0.802 - 0.941]**	**0.879 [0.809 - 0.955]**
	Heves	1.015 [0.926 - 1.113]	1.011 [0.921 - 1.109]	1.026 [0.937 - 1.124]	1.010 [0.929 - 1.098]	0.999 [0.915 - 1.092]	1.016 [0.927 - 1.114]
	Komárom-Esztergom	**1.106 [1.004 - 1.219]**	1.089 [0.988 - 1.202]	**1.104 [1.004 - 1.215]**	**1.093 [1.000 - 1.193]**	**1.106 [1.008 - 1.214]**	1.095 [0.994 - 1.206]
	Nógrád	1.105 [0.997 - 1.225]	**1.111 [1.002 - 1.232]**	**1.113 [1.005 - 1.233]**	**1.136 [1.035 - 1.248]**	**1.116 [1.011 - 1.233]**	**1.109 [1.000 - 1.229]**
	Pest	0.974 [0.912 - 1.041]	0.973 [0.911 - 1.040]	0.972 [0.910 - 1.037]	1.019 [0.961 - 1.081]	0.973 [0.913 - 1.036]	0.974 [0.912 - 1.040]
	Somogy	0.971 [0.884 - 1.067]	0.975 [0.887 - 1.072]	0.977 [0.891 - 1.073]	0.977 [0.898 - 1.063]	1.002 [0.916 - 1.095]	0.979 [0.892 - 1.076]
	Szabolcs-Szatmár-Bereg	1.080 [0.990 - 1.178]	1.084 [0.994 - 1.183]	**1.093 [1.004 - 1.191]**	**1.102 [1.020 - 1.191]**	1.070 [0.984 - 1.163]	1.080 [0.991 - 1.177]
	Jász-Nagykun-Szolnok	**0.855 [0.782 - 0.935]**	**0.858 [0.785 - 0.939]**	**0.858 [0.785 - 0.937]**	**0.910 [0.840 - 0.985]**	**0.855 [0.785 - 0.932]**	**0.862 [0.789 - 0.943]**
	Tolna	0.958 [0.857 - 1.070]	0.961 [0.860 - 1.074]	0.959 [0.859 - 1.069]	0.980 [0.888 - 1.081]	0.936 [0.841 - 1.040]	0.962 [0.862 - 1.074]
	Vas	0.996 [0.896 - 1.108]	0.994 [0.894 - 1.106]	1.002 [0.903 - 1.112]	1.051 [0.958 - 1.153]	1.013 [0.916 - 1.119]	0.992 [0.893 - 1.103]
	Veszprém	1.009 [0.920 - 1.106]	1.012 [0.923 - 1.109]	1.017 [0.930 - 1.113]	1.027 [0.947 - 1.115]	1.029 [0.944 - 1.122]	1.020 [0.932 - 1.117]
	Zala	0.903 [0.815 - 1.001]	0.909 [0.821 - 1.008]	0.922 [0.834 - 1.019]	0.945 [0.864 - 1.034]	0.927 [0.842 - 1.021]	0.905 [0.818 - 1.002]
Settlement type of GMP	Urban / Rural	0.987 [0.947 - 1.029]	0.985 [0.945 - 1.027]	0.979 [0.939 - 1.020]	0.972 [0.936 - 1.009]	0.992 [0.954 - 1.033]	0.989 [0.949 - 1.031]

		AstraZeneca	Janssen	Moderna	Pfizer	Sinopharm	Sputnik
Size of GMP (reference 1201-1600 patients)	≤ 800 patients	1.028 [0.934 - 1.132]	1.025 [0.931 - 1.129]	1.036 [0.942 - 1.139]	0.997 [0.915 - 1.086]	1.022 [0.931 - 1.120]	1.029 [0.935 - 1.133]
	801-1200 patients	1.033 [0.984 - 1.084]	1.035 [0.986 - 1.087]	1.042 [0.994 - 1.093]	1.007 [0.964 - 1.051]	1.035 [0.989 - 1.084]	1.037 [0.988 - 1.088]
	1601-2000 patients	1.017 [0.979 - 1.056]	1.021 [0.983 - 1.060]	1.021 [0.984 - 1.060]	1.014 [0.981 - 1.049]	1.016 [0.980 - 1.053]	1.019 [0.982 - 1.058]
	> 2000 patients	1.019 [0.978 - 1.062]	1.022 [0.981 - 1.066]	1.025 [0.984 - 1.067]	1.018 [0.981 - 1.057]	1.014 [0.975 - 1.055]	1.025 [0.983 - 1.068]
Age and sex of GP (reference < 65 years male)	< 65 years female	0.984 [0.948 - 1.022]	0.986 [0.949 - 1.024]	0.986 [0.951 - 1.024]	**0.963 [0.931 - 0.995]**	0.984 [0.949 - 1.020]	0.984 [0.948 - 1.022]
	≥ 65 years male	1.027 [0.983 - 1.072]	1.028 [0.985 - 1.074]	1.026 [0.983 - 1.071]	0.997 [0.959 - 1.036]	1.032 [0.991 - 1.076]	1.028 [0.985 - 1.073]
	≥ 65 years female	0.993 [0.946 - 1.043]	0.992 [0.944 - 1.041]	0.985 [0.939 - 1.034]	0.950 [0.909 - 0.992]	0.993 [0.948 - 1.040]	0.993 [0.946 - 1.042]
	Vacant	0.967 [0.909 - 1.027]	0.966 [0.908 - 1.027]	0.954 [0.898 - 1.014]	**0.921 [0.871 - 0.974]**	0.959 [0.904 - 1.017]	0.962 [0.905 - 1.022]
Relative education for GMP		0.674 [0.447 - 1.017]	0.698 [0.462 - 1.054]	**0.648 [0.432 - 0.973]**	**0.596 [0.413 - 0.860]**	**0.606 [0.409 - 0.898]**	0.693 [0.460 - 1.042]
Relative employment for GMP		0.911 [0.688 - 1.206]	0.894 [0.675 - 1.184]	0.919 [0.696 - 1.213]	0.911 [0.708 - 1.173]	0.910 [0.695 - 1.191]	0.882 [0.667 - 1.166]

Table S7. Mortality risk reduction among vaccinated adults by Cox proportional hazards regression models between 21 June 2021 and 15 August 2021 (adjusted hazard ratios with corresponding 95% confidence intervals, significant results in bold) in Hungary.

		AstraZeneca	Janssen	Moderna	Pfizer	Sinopharm	Sputnik
Vaccination	Fully / Not vaccinated	**0.317 [0.294 - 0.341]**	**0.707 [0.604 - 0.828]**	**0.438 [0.409 - 0.469]**	**0.384 [0.370 - 0.399]**	**0.312 [0.297 - 0.328]**	**0.221 [0.202 - 0.242]**
Age in years		**1.092 [1.090 - 1.094]**	**1.092 [1.090 - 1.094]**	**1.092 [1.091 - 1.094]**	**1.095 [1.093 - 1.096]**	**1.094 [1.092 - 1.096]**	**1.092 [1.091 - 1.094]**
Sex	Female / Male	**0.579 [0.552 - 0.607]**	**0.582 [0.554 - 0.612]**	**0.597 [0.570 - 0.626]**	**0.608 [0.586 - 0.631]**	**0.589 [0.564 - 0.615]**	**0.574 [0.546 - 0.602]**
Exemption certificate	Yes / No	**1.747 [1.603 - 1.905]**	**1.737 [1.585 - 1.904]**	**1.717 [1.576 - 1.872]**	**1.602 [1.498 - 1.713]**	**1.634 [1.507 - 1.773]**	**1.729 [1.580 - 1.893]**
Diabetes mellitus	Yes / No	**1.329 [1.247 - 1.417]**	**1.297 [1.211 - 1.390]**	**1.340 [1.259 - 1.427]**	**1.349 [1.287 - 1.414]**	**1.327 [1.253 - 1.405]**	**1.340 [1.253 - 1.432]**
Ischemic heart disease	Yes / No	**1.457 [1.342 - 1.582]**	**1.437 [1.316 - 1.568]**	**1.413 [1.303 - 1.532]**	**1.381 [1.300 - 1.466]**	**1.491 [1.386 - 1.605]**	**1.484 [1.363 - 1.616]**
COPD	Yes / No	**1.848 [1.705 - 2.003]**	**1.821 [1.672 - 1.984]**	**1.850 [1.709 - 2.001]**	**1.751 [1.648 - 1.861]**	**1.822 [1.692 - 1.962]**	**1.807 [1.660 - 1.965]**
Asthma	Yes / No	not computable	0.703 [0.099 - 5.001]	not computable	0.427 [0.060 - 3.032]	not computable	not computable
Hypertension	Yes / No	**1.088 [1.030 - 1.149]**	**1.086 [1.026 - 1.150]**	**1.064 [1.008 - 1.123]**	0.994 [0.951 - 1.039]	1.044 [0.992 - 1.098]	**1.079 [1.021 - 1.140]**
Cancer	Yes / No	**4.907 [4.603 - 5.232]**	**5.273 [4.931 - 5.639]**	**4.563 [4.283 - 4.862]**	**3.466 [3.302 - 3.638]**	**4.133 [3.892 - 4.389]**	**5.111 [4.783 - 5.463]**
Chronic liver disease	Yes / No	**16.457 [12.052 - 22.471]**	**17.440 [12.668 - 24.011]**	**15.849 [11.563 - 21.724]**	**12.672 [9.593 - 16.739]**	**19.799 [15.065 - 26.022]**	**16.569 [11.933 - 23.007]**
End-stage renal disease	Yes / No	**3.668 [2.935 - 4.584]**	**3.293 [2.561 - 4.235]**	**3.374 [2.718 - 4.189]**	**3.426 [2.975 - 3.946]**	**3.491 [2.820 - 4.322]**	**3.304 [2.570 - 4.248]**
Location of GMP by county (reference Budapest)	Baranya	**0.828 [0.717 - 0.957]**	**0.830 [0.713 - 0.966]**	**0.824 [0.714 - 0.951]**	**0.872 [0.782 - 0.973]**	**0.848 [0.744 - 0.965]**	**0.840 [0.725 - 0.974]**
	Bács-Kiskun	0.901 [0.792 - 1.025]	0.917 [0.801 - 1.048]	0.924 [0.815 - 1.047]	0.953 [0.862 - 1.053]	**0.885 [0.787 - 0.996]**	0.907 [0.796 - 1.034]
	Békés	**0.841 [0.730 - 0.970]**	**0.821 [0.707 - 0.954]**	**0.835 [0.725 - 0.961]**	0.982 [0.882 - 1.093]	**0.829 [0.728 - 0.945]**	**0.845 [0.732 - 0.976]**
	Borsod-Abaúj-Zemplén	0.938 [0.830 - 1.059]	0.929 [0.817 - 1.057]	0.932 [0.826 - 1.052]	1.006 [0.913 - 1.108]	0.905 [0.808 - 1.013]	0.935 [0.825 - 1.059]
	Csongrád-Csanád	0.911 [0.798 - 1.040]	0.929 [0.810 - 1.066]	0.930 [0.815 - 1.062]	0.987 [0.893 - 1.092]	0.898 [0.796 - 1.013]	0.919 [0.804 - 1.051]
	Fejér	0.888 [0.770 - 1.025]	0.924 [0.795 - 1.074]	0.938 [0.816 - 1.078]	0.977 [0.876 - 1.089]	0.917 [0.806 - 1.044]	0.930 [0.805 - 1.075]
	Győr-Moson-Sopron	1.072 [0.935 - 1.229]	1.097 [0.949 - 1.268]	1.075 [0.938 - 1.232]	1.089 [0.979 - 1.212]	1.032 [0.910 - 1.170]	1.053 [0.914 - 1.212]
	Hajdú-Bihar	**0.829 [0.727 - 0.944]**	**0.854 [0.746 - 0.978]**	**0.858 [0.754 - 0.975]**	**0.867 [0.783 - 0.961]**	**0.841 [0.746 - 0.947]**	**0.859 [0.753 - 0.981]**
	Heves	0.910 [0.784 - 1.055]	0.922 [0.789 - 1.078]	0.941 [0.813 - 1.088]	0.945 [0.842 - 1.061]	**0.870 [0.758 - 0.997]**	0.910 [0.782 - 1.060]
	Komárom-Esztergom	0.948 [0.808 - 1.112]	0.906 [0.762 - 1.076]	0.902 [0.768 - 1.058]	1.021 [0.902 - 1.156]	0.885 [0.763 - 1.026]	0.953 [0.810 - 1.121]
	Nógrád	0.874 [0.733 - 1.041]	0.912 [0.762 - 1.091]	0.900 [0.758 - 1.069]	0.980 [0.857 - 1.120]	0.885 [0.753 - 1.040]	0.909 [0.762 - 1.085]
	Pest	0.977 [0.880 - 1.085]	1.024 [0.917 - 1.142]	1.014 [0.916 - 1.122]	1.042 [0.962 - 1.128]	0.945 [0.860 - 1.038]	0.990 [0.890 - 1.102]
	Somogy	0.973 [0.840 - 1.127]	0.982 [0.842 - 1.147]	0.966 [0.834 - 1.118]	1.014 [0.905 - 1.136]	0.996 [0.872 - 1.138]	0.948 [0.815 - 1.103]
	Szabolcs-Szatmár-Bereg	**0.859 [0.745 - 0.991]**	0.906 [0.782 - 1.050]	0.927 [0.808 - 1.065]	0.960 [0.861 - 1.070]	0.896 [0.788 - 1.020]	0.903 [0.783 - 1.042]
	Jász-Nagykun-Szolnok	0.985 [0.863 - 1.124]	1.002 [0.874 - 1.150]	1.042 [0.917 - 1.185]	1.013 [0.912 - 1.125]	0.948 [0.839 - 1.070]	1.003 [0.877 - 1.147]
	Tolna	0.920 [0.774 - 1.094]	0.926 [0.772 - 1.111]	0.972 [0.822 - 1.148]	0.999 [0.878 - 1.137]	0.883 [0.755 - 1.033]	0.886 [0.741 - 1.060]
	Vas	0.907 [0.765 - 1.076]	0.929 [0.777 - 1.111]	0.911 [0.770 - 1.078]	0.976 [0.858 - 1.110]	0.931 [0.800 - 1.083]	0.936 [0.788 - 1.111]
	Veszprém	0.882 [0.759 - 1.024]	0.867 [0.740 - 1.016]	0.867 [0.748 - 1.005]	0.971 [0.866 - 1.088]	**0.829 [0.722 - 0.951]**	**0.841 [0.720 - 0.981]**
	Zala	**0.776 [0.658 - 0.916]**	**0.793 [0.667 - 0.944]**	**0.826 [0.703 - 0.971]**	**0.877 [0.774 - 0.992]**	**0.813 [0.701 - 0.942]**	**0.805 [0.681 - 0.951]**
Size of GMP (reference 1201-1600 pa-	≤ 800 patients	0.942 [0.809 - 1.098]	0.944 [0.807 - 1.105]	0.949 [0.817 - 1.101]	0.945 [0.841 - 1.062]	1.030 [0.900 - 1.178]	0.998 [0.858 - 1.161]
	801-1200 patients	0.985 [0.912 - 1.063]	0.965 [0.891 - 1.045]	0.955 [0.885 - 1.031]	0.996 [0.939 - 1.056]	0.990 [0.924 - 1.062]	0.990 [0.916 - 1.070]

		AstraZeneca	Janssen	Moderna	Pfizer	Sinopharm	Sputnik
tients)	1601-2000 patients	1.004 [0.945 - 1.066]	0.994 [0.933 - 1.058]	0.992 [0.935 - 1.052]	0.987 [0.941 - 1.034]	0.987 [0.934 - 1.044]	1.002 [0.942 - 1.065]
	> 2000 patients	1.004 [0.940 - 1.073]	0.992 [0.926 - 1.063]	0.990 [0.928 - 1.056]	1.022 [0.971 - 1.076]	1.030 [0.970 - 1.094]	0.996 [0.931 - 1.065]
Settlement type of GMP	Urban / Rural	0.982 [0.919 - 1.049]	1.000 [0.933 - 1.072]	0.981 [0.919 - 1.048]	0.980 [0.931 - 1.033]	0.987 [0.929 - 1.049]	0.995 [0.930 - 1.065]
Age and sex of GP (reference < 65 years male)	< 65 years female	0.974 [0.918 - 1.034]	0.981 [0.922 - 1.045]	0.980 [0.924 - 1.039]	0.977 [0.933 - 1.023]	0.972 [0.920 - 1.027]	0.974 [0.916 - 1.035]
	≥ 65 years male	0.967 [0.902 - 1.037]	0.975 [0.906 - 1.049]	0.986 [0.920 - 1.056]	0.960 [0.909 - 1.014]	0.978 [0.917 - 1.042]	0.990 [0.922 - 1.063]
	≥ 65 years female	0.979 [0.905 - 1.058]	1.001 [0.923 - 1.086]	0.988 [0.915 - 1.066]	0.964 [0.908 - 1.024]	0.982 [0.914 - 1.054]	1.014 [0.937 - 1.097]
	Vacant	1.066 [0.971 - 1.170]	1.060 [0.962 - 1.167]	1.032 [0.940 - 1.133]	0.972 [0.901 - 1.048]	1.074 [0.985 - 1.171]	1.048 [0.952 - 1.153]
Relative education for GMP		0.623 [0.325 - 1.197]	0.651 [0.329 - 1.288]	**0.463 [0.242 - 0.884]**	**0.434 [0.261 - 0.723]**	**0.484 [0.265 - 0.883]**	0.596 [0.306 - 1.160]
Relative employment for GMP		0.781 [0.503 - 1.212]	0.760 [0.481 - 1.201]	0.832 [0.537 - 1.288]	0.807 [0.569 - 1.144]	0.861 [0.572 - 1.295]	0.779 [0.498 - 1.220]
